# Massive *Cryptosporidium* infections and chronic diarrhea in HIV-negative patients

**DOI:** 10.1007/s00436-019-06302-0

**Published:** 2019-04-12

**Authors:** Łukasz Pielok, Szymon Nowak, Matylda Kłudkowska, Krystyna Frąckowiak, Łukasz Kuszel, Paweł Zmora, Jerzy Stefaniak

**Affiliations:** 1 Department and Clinic of Tropical and Parasitic Diseases, Poznań University of Medical Sciences, Przybyszewskiego Street, 60-355 Poznań, Poland; 2 Central Laboratory of Microbiology, H. Święcicki University Hospital, Poznań, Poland; 3 Department of Medical Genetics, Poznań University of Medical Sciences, Poznań, Poland; 4 Institute of Bioorganic Chemistry Polish Academy of Sciences, Poznań, Poland

**Keywords:** *Cryptosporidium* spp., Cryptosporidiosis, Diarrhea, Chronic diarrhea, Cryptitis, Nitazoxanide, Immunocompetent

## Abstract

Protozoa of the genus *Cryptosporidium* are common parasites of domestic and wild animals—mammals, birds, reptiles, and fishes. The invasive forms are thick-walled oocysts, which can be present in water supplies, on fruits, vegetables, or in the soil contaminated with feces. In this work, we describe three cases of middle-aged persons with massive *Cryptosporidium hominis* infection and chronic diarrhea with no immunological abnormalities and no history of previous travels to tropical countries. The lesions discovered during colonoscopy within the large intestine–cryptitis and the histopathological changes were related to massive cryptosporidiosis. All these statements indicate necessity of parasitological stool examination in cases with chronic diarrhea in which no etiological agents are detected, but not only in HIV positive individuals. Parasite’s eradication leads to symptom disappearance as well as improvement of histopathological mucosa alterations.

## Introduction

Protozoa of the genus *Cryptosporidium* are common parasites of domestic and wild animals—mammals, birds, reptiles, and fishes (Zehedi et al. 2015; Osman et al. 2017; Yu et al. 2018). The invasive forms for all hosts are thick-walled oocysts, strongly resistant for chlorine disinfection, which can be present in water supplies, on fruits, vegetables, or in the soil contaminated with feces (Toledo et al. 2017; Squire and Ryan 2017; Domenech et al. 2018). The most important way of transmission of these parasites is the fecal-oral route, by swallowing oocysts with contaminated food or nontreated water or during recreational water events (lakes, rivers, and swimming pools) (Hall et al. 2017; Fill et al. 2017; Hlavsa et al. 2017). The presence of *Cryptosporidium* spp. and *Giardia intestinalis* in waste water is a main concern because water reuse for irrigation can jeopardize human health (Domenech et al. 2018). The direct way of transmission from person to person of these apicomplexan parasites is also possible, that is why human, especially asymptomatic carrier, becomes a possible source of infection for his surroundings, creating a serious epidemiological threat.

*Cryptosporidium* trophozoites exist in the enterocytes and are responsible for their damage causing gastrointestinal symptoms such as watery diarrhea and abdominal cramps (Bouzid et al. 2013). The pathologic process in an immunocompetent host is usually self-limiting, but sometimes, protracted infections can be present. In such cases, chronic diarrhea lasts longer than 4–6 weeks (Sandhu and Surawicz 2012). Persistent intestinal disorders are present mainly in persons suffering from different types of immunodeficiency. Cryptosporidiosis is classically reported in patients with acquired immunodeficiency syndrome and emerged as a cause of persistent diarrhea in solid transplant patients (Kaniyarakkal et al. 2016; DuPont 2016). Cryptosporidiosis is also a late post-transplant infection that can disseminate to biliary ducts or lungs (Lantermier et al. 2017). In HIV-infected patients, it is an opportunistic infection—an indicator of full symptomatic AIDS (Shrivastava et al. 2017a, 2017b).

In this work we describe three cases of middle-aged persons with massive *Cryptosporidium* spp. infection and chronic diarrhea with no immunological abnormalities.

## Case 1

Forty-six-year-old male admitted to the Clinic of Tropical and Parasitic Diseases, Poznan, Poland, because of persistent low-grade fever, lymphadenopathy, joint pains, and watery diarrhea lasting for 5 weeks. Prior to the admission, he was hospitalized in the Internal Ward, but no tentative diagnosis was established, except chronic tonsillitis (he was classified among surgery treatment). Previously, he was also suffered from skin *Streptococcus pyogenes* and *Staphylococcus aureus* mixed infection. No history of previous travels.

**Fig. 1:**
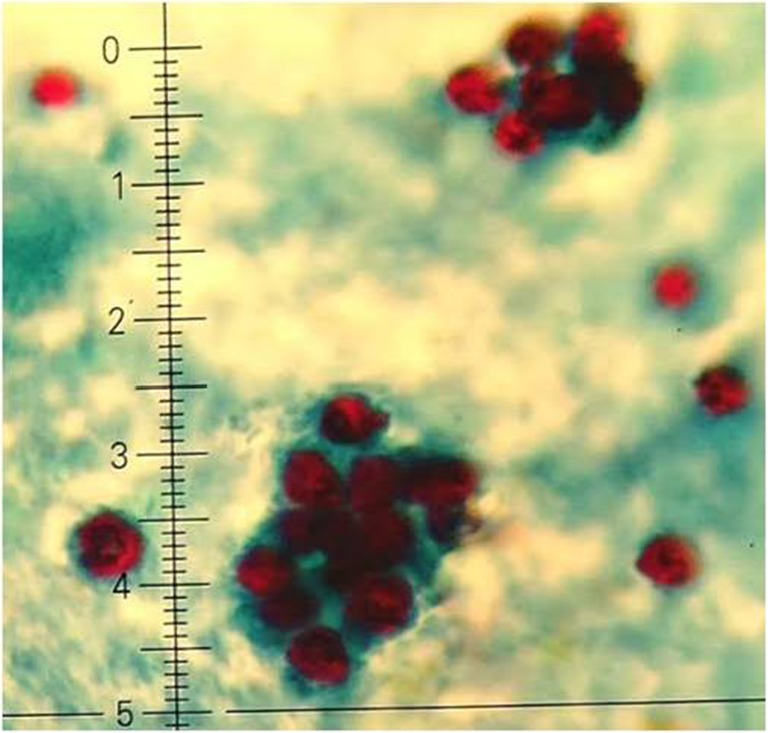
Microscopical analysis with Ziehl–Neelsen modified staining revealed numerous oocysts of Cryptosporidium spp. in the patient’s feces

On admission to the Clinic, he was afebrile. Physical examination revealed presence of cervical and axillar lymphadenopathy and increased bowel movements. Blood tests confirmed an internal inflammation (elevated levels of ESR 36 mm/h, CRP 31.8 mg/l, WBC 9.17 G/l). Because of diarrhea, stool examination was performed. Bacteriological culture according to enteropathogenic bacteria (*Salmonella* spp*.*, *Shigella* spp., *E.coli* ETEC, *Yersinia* spp., *Campylobacter* spp*.*, *Clostridium difficile*) was negative. However, the modified Ziehl–Neelsen staining smears revealed presence of huge amount of *Cryptosporidium* spp. Oocysts, i.e., 20–50 oocysts/10 fields (magnification 1000×), which according to Castro-Hermida et al. (2001) was classified as a massive infection (Fig. 1). Moreover, elevated fecal calprotectin concentration was detected. Fecal occult blood test (FOBT) was also positive. Electrophoresis showed increased levels of α2-, β2-, and γ-globulins (15.6/6.9/16.8%) and shortage of albumins (47%).

Because of the massive cryptosporidiosis, the immunodeficiency diagnosis was initiated. Architect CMIA HIV test was negative, HIV-RNA (HIV Cobas TaqScreen MPX version 2.0-Roche) was also negative, normal CD4 count (507/mm3), *Treponema* RPR test negative, ELISA *Toxoplasma gondii* (IgM and IgG) negative, ELISA *Toxocara* IgG negative, HBsAg absent, anti-HCV absent, normal immunoglobulin levels (IgA 1.89 g/l, IgG 11.43 g/l, IgM 0.79 g/l). Colonoscopy (after the patient’s permission) detected numerous small afts and shallow ulcers as well as mucosa scarifications.

**Fig. 2:**
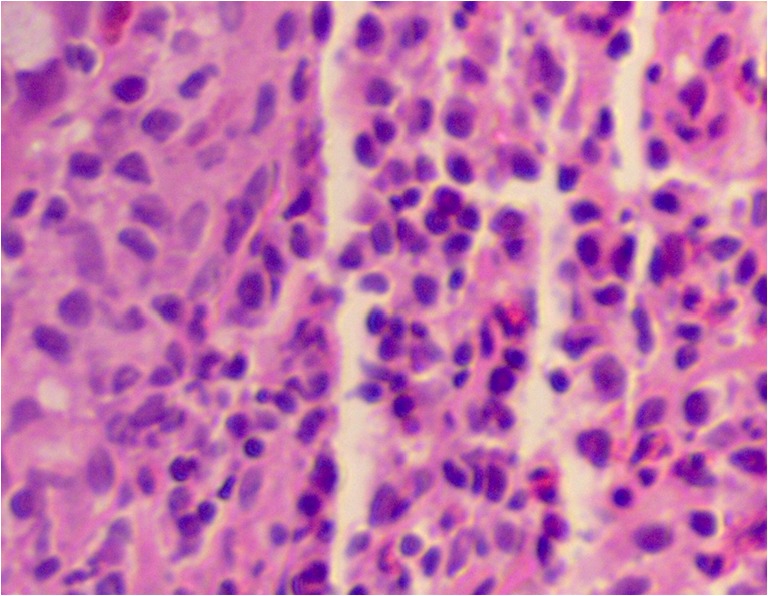
Large intestine mucosa infiltrated by eosinophils and neutrophils (hematoxylin and eosine staining)

The histopathological preparations showed incorrect large intestine mucosa architecture with persistent inflammation within submucosa caused by eosinophils and disseminated in lamina propria neutrophils (cryptitis, Fig. 2).

The patient was treated with tilbroquinolonum, trimethoprim/sulphametoxazole (2 × 960 mg), *Saccharomyces boulardii*, spiramycin (3 × 3.0 mln u.). The patient improved and the parasitic stool test on the last day (after 2 weeks of hospitalization) was negative. Patient was admitted to the Clinic once again after 2 weeks because of relapse of the symptoms (15–20 watery stools, abdominal pain, fever up to 39 °C). The stool examination revealed massive *Cryptosporidium* spp. infection, i.e., 20–30 oocysts/10 fields (magnification 1000×, Castro-Hermida et al. 2001). The presence of other parasites was excluded. Combined therapy with Pyrimethamine (1 × 75 mg), Azythromycin (1 × 500 mg), and Nitazoxanide (2 × 500 mg) was initiated. After 10-day treatment, *Cryptosporidium* eradication was obtained with confirmation after 3 weeks. In control colonoscopy, no signs of inflammatory process were found.

## Case 2

Fifty-six-year-old male hospitalized for 14 days in the Clinic of Tropical and Parasitic Diseases, Poznan, Poland, because of persistent diarrhea, 4–5 watery stools without blood and mucus and fever up to 39 °C. The symptoms appeared 1 year before the admission to the hospital. The patient was treated symptomatically and empirically with amoxicillin, ciprofloxacin, cefuroxime with periodic improvement. He also lost 10 kg of weight. His medical history of chronic diseases and previous travels was unremarkable.

**Fig. 3:**
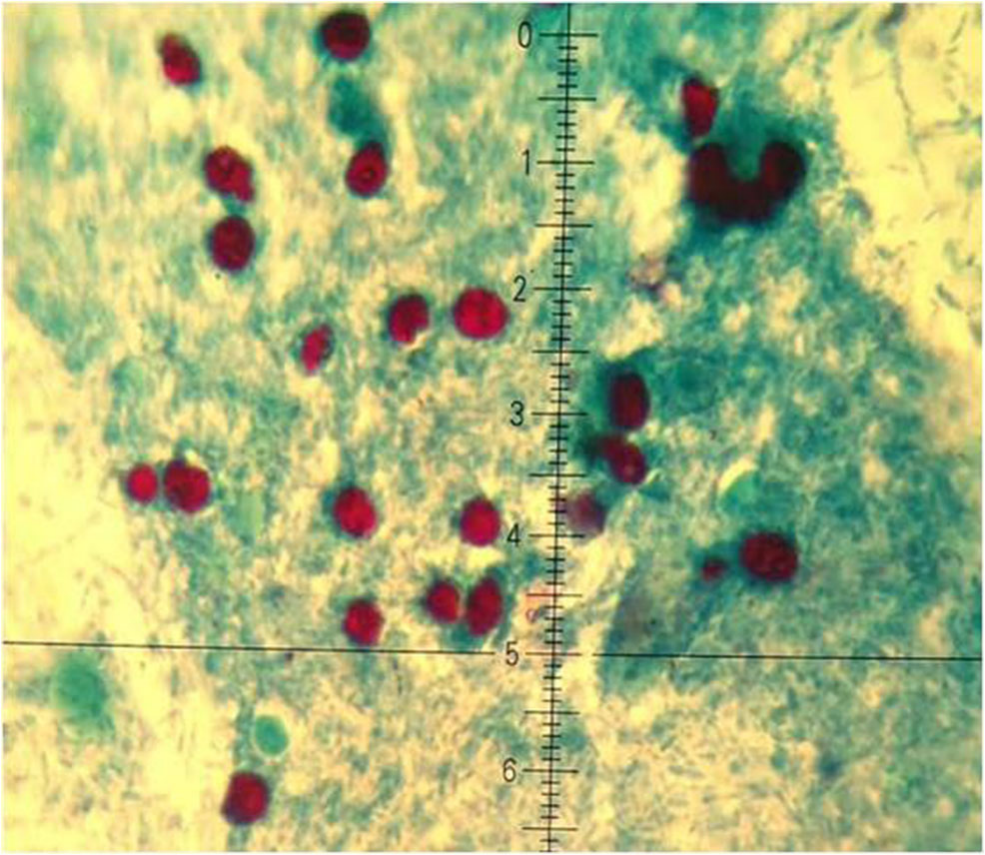
Cryptosporidium spp. oocyts (modified Ziehl–Neelsen staining)

The physical examination performed on admission revealed presence of numerous enlarged, solid cervical lymphatic nodes, tenderness to palpation in the left subcostal region, and intensive bowel movements. Laboratory findings confirmed acute inflammatory response: increased level of CRP (79.3 ng/l, ESR 47 mm/h, leukocytosis 16.73 G/l), monocytosis (8.4%), neutrocytosis (81.3%), and thrombocytosis (672 G/l). Bacteriological stool tests excluded *Salmonella*, *Shigella*, *Escherichia coli*, and *Yersinia* spp. infection. Repeated parasitological stool examinations (modified Ziehl–Neelsen staining) revealed presence of numerous *Cryptosporidium* spp. oocysts, i.e., 20–50 oocysts/10 fields (magnification 1000×; Fig. 3) and elevated level of WBCs and RBCs. High level of fecal calprotectin (895 μg/l) in the stool sample indicated acute bowel inflammation. FOBT was positive. Possible immunodeficiency was excluded, as in the case no. 1.

Colonoscopy (after the patient’s permission) performed during hospitalization revealed presence intensive inflammatory mucosal lesions, numerous polyps narrowing the bowel bore, presence of stool-purulent content, and different-shape ulcers (cryptitis). CT scans showed the extraperitoneum lymphadenopathy and intestine wall inflammation (mucosa thickening). Histopathologic sections showed incorrect lamina propria architecture with cryptitis. Chronic, severe inflammation was detected in submucosa. The most abundant type of cells was eosinophils. Disseminated neutrophils, mucosal ulcers covered with granular, and fibrinous changes were also observed.

Combined therapy with trimethoprim/sulphametoxazole and rifaximin was initiated together with symptomatic treatment (20% albumins, iron supplementation, anti-diarrheal agents). The improvement of the clinical condition was observed, and the patient was discharged home.

After 2 months, the patient was admitted to the Clinic for follow-up. Several parasitic stool examinations were negative. Colonoscopy showed only presence of pseudopolyp and only slight mucosal changes. The histopathological section without abnormalities. Complete normalization of laboratory parameters (CRP-8.7 ng/l, ESR–7 mm/h, WBC-10.1 G/l, PLT–473 G/l, iron 82 μg/dl, TIBC 396 μg/dl) was observed. Parasitological stool examination revealed *Cryptosporidium* eradication.

## Case 3

Forty-six-year-old woman admitted to the hospital for the differential diagnosis of chronic diarrhea. From 1 month, she had several watery, sometimes bloody stools with high fever up to 39°. Laboratory results showed high CRP level (37.1 ng/l), increased ESR (33 mm/h), WBC (9.1G/l). USG of the abdomen cavity did not reveal any abnormalities. Bacteriological stool culture detected *Campylobacter jejuni*. The patient did not consent to the colonoscopy.

Parasitic stool examination showed massive infection with *Cryptosporidium* spp. oocysts, i.e., 10–40 oocysts/10 fields (magnification 1000×, Castro-Hermida et al. 2001). High fecal calprotectin level was also detected (> 1000 μg/g). The FOBT was positive. The immunodeficiency was excluded like in the previous cases. The patient was treated with trimethoprim/sulphametoxazole (2 × 960 mg), azythromycin (1 × 500 mg), rifaximin (2 × 400 mg). After 10-day-treatment symptoms diminished, stool examinations were negative, and the patient was discharged home.

During follow-up visit in out-patient department, she reported numerous loose stools. Parasitological examinations of the stool samples discovered *Cryptosporidium* oocysts. Treatment with nitazoxanide was initiated (3 × 500 mg for 1 week and 2 × 500 mg for 3 days). After the treatment, parasite eradication was obtained.

## Genotyping of *Cryptosporidium*

DNA was extracted from stool samples using a commercial DNA extraction kit (NucleoSpin® Tissue, Macherey-Nagel GmbH & Co. KG, Düren, Germany) according to the support protocol for genomic DNA stool. To increase the yield of the DNA isolation, five cycles of freezing (− 80 °C) and thawing (50 °C) of the stool samples were performed. Because of the consistency of the fecal samples, the isolation of each sample was carried out in duplicate, and the DNA samples were concentrated using Eppendorf Concentrator plus (Eppendorf AG, Hamburg, Germany). To identify the *Cryptosporidium* species, the multiplex allele specific polymerase chain reaction (MAS-PCR) was performed according to Gile et al. with some modifications. (Gile et al. 2002). Briefly, the PCR was performed in a total volume of 20 μl, consisting of 200 ng isolated DNA, 1.25 U of DreamTaq DNA polymerase (Thermo Fisher Scientific), 2 μl of DreamTaq Buffer (10×), 10 pmol of dNTPs, and 20 pmol of forward (CINF) primer and 10 pmol of reverse (CINR, 1R, 2R) primers. The PCR conditions were initial denaturation at 95 °C for 5 min, followed by 35 cycles of denaturation at 95 °C for 30 s, annealing at 50 °C for 30 s, and elongation at 72 °C for 1 min, with final elongation at 72 °C for 10 min. Amplified products were subjected to electrophoreses in a 2% agarose gel. The results were confirmed in three independent reaction sets.

**Fig. 4:**
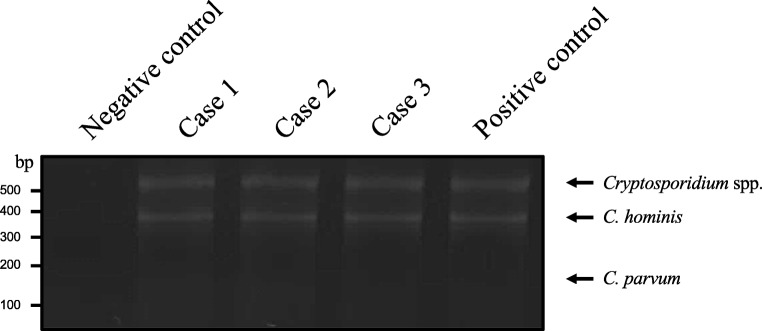
Genotyping of *Cryptosporidium* spp.

The MAS-PCR, used for the identification of *Cryptosporidium* species, amplifies the dihydrofolate reductase (DHFR) gene with one sense and three antisense primers (CINF and CINR, 1R, 2R, respectively). Primer-pairs CINF and CINR amplify a 575 bp region of the DHFR gene, specific for *Cryptosporidium* spp., and simultaneously confirm the infection with this pathogen. Additionally, the primer set CINF and 1R amplifies a 357 bp region, specific for *C. hominis*, while the primer-pair CINF and 2R gives the product with 190 bp size, specific for *C. parvum*. In all three cases, the 575 bp band was detected, confirming the infection with *Cryptosporidium* spp. Additionally, the band with 357 bp size was observed in all three cases, suggesting the infection with *Cryptosporidium hominis* (Fig. 4). The specific band for *C. parvum* was not detected in any sample. As a negative control, we used the stool sample from a patient without cryptosporidiosis, and any band was not observed. The stool from the patient with previously diagnosed cryptosporidiosis was taken as a positive control (Fig. 4).

## Discussion

Chronic diarrhea lasts longer than three and is rarely caused by the infectious agents (Sandhu and Surawicz 2012). Protozoa of the genus *Cryptosporidium* are considered as one of the etiologic agents of persistent diarrheal illness in immunocompromised patients (Clemente et al. 2000; Werneck-Silva and Bedin Prado 2009). However, they are more often detected also in HIV-negative humans, as a cause of travelers’ diarrhea, in immunocompetent children or in man infected with the hedgehog genotype (Kłudkowska et al. 2017; Tallant et al. 2016; Kváč et al. 2014).

The main clinical symptoms observed in the infected patients are watery diarrhea, abdominal pains, and mild fever. Interestingly, non-intestinal sequelae of cryptosporidiosis can be also present, i.e., arthropathies, stiffness in the lumbo-sacralis region, eye pains, and headaches (Hunter et al. 2014). In one of the described patients painful, inflamed joints were also observed. According to the literature, the extraintestinal signs occur even in 30% of infected individuals (Lopez-Velez et al. 1995). Moreover, hematogenous *Cryptosporidium* spread with the blood vessels was observed (Gentile et al. 1987) and negative changes in the liver and the heart were detected. The intestinal parasitic infections may bring about functional impairments of internal organs leading to long-term consequences (Beier et al. 2006). Cryptosporidium oocysts were found in the trachea, sputum, and sinus aspirates. Cryptosporidiosis in the upper respiratory tract might cause inflammation in the sinuses, larynx, and nasal mucosa (Abdali et al. 2018). However, all these complications were detected in HIV-infected individuals. The clinical course is mostly self-limiting. However, in cases of massive or mixed infections, persistent symptoms are observed, and patients require combined anti-parasitic and also anti-bacterial treatment.

Massive cryptosporidiosis described in our three patients was not related to previous travels into tropical regions. They also deny having contact with any animals and had normal diet. Moreover, the stools of other roommates were examined and were parasitologically negative. Immunodeficiency diagnostics did not reveal any abnormalities. The lesions discovered during colonoscopy were related to massive cryptosporidiosis, since long-lasting and severe infection leads to lamina propria damage (Abdou et al. 2013). In the literature, you can find descriptions of endoscopic and histopathological changes in the stomach mucosa caused by *Cryptosporidium* spp., but they are mainly referred to immunocompromised persons (Clemente et al. 2000; Mohammadpour et al. 2016). Histopathologic features associated to *Cryptosporidium* infection include stomach infiltrates, villus lining in the duodenum, cryptitis caused by massive eosinophils concentration, and epithelial apoptosis in the colon (Werneck-Silva and Bedin Prado 2009). However, most of these findings concern to the HIV-positive patients.

The cases described in our study indicate that coccidian parasites might be responsible for gastrointestinal pathology resulting with chronic diarrhea also in HIV-negative persons. Massive infections should be treated because they lead to huge intestine damage, which could be responsible for prolonged even life-threatening symptoms especially in children, teenagers, and malnourished individuals. Moreover, they can influence on gut microbiota. The current therapeutic options for cryptosporidiosis are limited and only partially effective. In two out of three described cases, the treatment with trimethoprim/sulphametoxazole was insufficient. Nitazoxanide and azithromycin have better effectiveness for cryptosporidiosis treatment in humans (Lee et al. 2017).

To sum up, *Cryptosporidium* infection must be taken under consideration in differential diagnosis of persistent diarrhea in individuals without HIV infection. Additionally, it should be highlighted that persistent *Cryptosporidium hominis* infection can be responsible for enhanced lamina propria and submucosa alterations within large intestine, which strictly connects to prolonged intestinal symptoms in patients without HIV infection. Moreover, *Cryptosporidium* infection may also lead to extraintestinal abnormalities, i.e., extraperitoneal lymphadenopathy as well as generalized arthritis, and thus, patients suffering from such symptoms should be carefully examined to detect coccidian parasites. All these statements indicate that parasite’s eradication leads to symptom disappearance as well as improvement of histopathological mucosa alterations.
